# Supplementary material for: Convergence of Alcohol Consumption and Dietary Quality in US Adults Who Currently Drink Alcohol: An Analysis of Two Core Risk Factors of Liver Disease
Source: Nutrients. 2024 Nov 13;16(22):3866. doi: 10.3390/nu16223866 (PMC11597591; doi:10.3390/nu16223866)
Supplement: Supplementary file 1 [file nutrients-16-03866-s001.zip › nutrients-3245129-supplementary.pdf]

Supplemental Table S1 Average Calories, Added sugar, and Alcohol Intake from Alcoholic Beverages Among Each Type of Alcoholic Beverage Consumers.

	Wine only mean±se[1]	Beer only mean±se[1]	Liquor or cocktails only mean±se[1]	Multiple types[2] mean±se[1]	*P* value
**Nutrients intake from alcoholic beverage during the past 24 hours**					
Energy, kcal	157±10.0	287±17.5**	301±30.1**	439.5±34.5**	<0.001
Added sugar, gram	3.9±1.1	1.8±0.4	12.8±1.5**	12.9±2.3*	<0.001
Alcohol, gram	18.4±1.1	28.8±1.8**	34.3±3.8**	46.9±3.2**	<0.001

[1] Results were obtained after adjusting sample weights and complex study design.

[2] Individuals consumed multiple types of alcoholic beverage during the past 24 hours, including beer and wine, beer and liquor, wine and liquor or beer, wine and liquor.

*, $p < 0.05$; **, $p<0.001$ when compared to wine only.

P value presents the heterogeneity of nutrients intake from alcoholic beverage across each type of alcoholic beverage consumers by using Wald test.

Supplementary Table S2 The Association Between Amount of Alcohol Use and Healthy Eating Index (HEI) Among All Current Alcohol Drinkers

	Total HEI score		
**Amount of alcohol use**	Mean±SE	aDiff±SE	*P* value
Current light[1]	52.6±0.7	ref	
Current moderate/heavy	51.2±0.8	0.58±0.94	0.541
Remote history of moderate/heavy[2]	46.5±1.1	-3.26±1.24	0.014

aDiff represents the covariate-adjusted mean difference between light alcohol use and other levels. Regression models were adjusted for age, gender, race, poverty-to-income ratio, status of cigarette use, physical activity, medical conditions, and the presence of metabolic syndrome.

[1] Current light drinking defined as without past history of heavy drinking by ALQ151

[2] Remote history of heavy drinking defined as current light drinking but positive ALQ151

Abbreviations: SE, standard error

Supplementary Table S3 The Association Between Type of Alcoholic Beverage and Healthy Eating Index Scores Among Alcohol Drinkers In Current Heavy Alcohol Drinkers and Current Light Drinkers Without Past History of Heavy Alcohol Use (n=1765)

	**Model 1**		**Model 2**		**Model 3**	
	adj. β	95% CI	adj. β	95% CI	adj. β	95% CI
**Main predictor**						
Type of alcoholic beverage intake						
Wine only	1		1		1	
Beer only	-5.33**	(-7.64, -3.01)	-4.00*	(-6.29, -1.71)	-3.97*	(-6.28, -1.66)
Liquor or cocktails only	-1.03	(-4.06, 2.00)	0.06	(-2.89, 3.01)	0.09	(-2.90, 3.07)
Multiple types	-1.54	(-5.12, 2.03)	-1.16	(-4.52, 2.20)	-1.17	(-4.55, 2.21)

Model 1 was adjusted for age, sex, race, and poverty-to-income ratio.
Model 2 was adjusted for covariates in Model 1 and status of cigarettes and amount of alcohol use, physical activity, and medical conditions.
Model 3 was adjusted for covariates in Model 2, and the presence of metabolic syndrome.
*, $p < 0.05$; **, $p<0.01$ when compared to wine only.
